# Incidence of acute pericarditis after pulsed-field ablation for the treatment of atrial fibrillation

**DOI:** 10.1093/europace/euae180

**Published:** 2024-06-28

**Authors:** Corinne Isenegger, Rebecca Arnet, Fabian Jordan, Marc Salis, Sven Knecht, Philipp Krisai, Gian Völlmin, David Spreen, Stefan Osswald, Christian Sticherling, Michael Kühne, Patrick Badertscher

**Affiliations:** Department of Cardiology, University Hospital Basel, University of Basel, Petersgraben 4, 4031 Basel, Switzerland; Cardiovascular Research Institute Basel, University Hospital Basel, University of Basel, Petersgraben 4, 4031 Basel, Switzerland; Department of Cardiology, University Hospital Basel, University of Basel, Petersgraben 4, 4031 Basel, Switzerland; Cardiovascular Research Institute Basel, University Hospital Basel, University of Basel, Petersgraben 4, 4031 Basel, Switzerland; Department of Cardiology, University Hospital Basel, University of Basel, Petersgraben 4, 4031 Basel, Switzerland; Cardiovascular Research Institute Basel, University Hospital Basel, University of Basel, Petersgraben 4, 4031 Basel, Switzerland; Department of Cardiology, University Hospital Basel, University of Basel, Petersgraben 4, 4031 Basel, Switzerland; Cardiovascular Research Institute Basel, University Hospital Basel, University of Basel, Petersgraben 4, 4031 Basel, Switzerland; Department of Cardiology, University Hospital Basel, University of Basel, Petersgraben 4, 4031 Basel, Switzerland; Cardiovascular Research Institute Basel, University Hospital Basel, University of Basel, Petersgraben 4, 4031 Basel, Switzerland; Department of Cardiology, University Hospital Basel, University of Basel, Petersgraben 4, 4031 Basel, Switzerland; Cardiovascular Research Institute Basel, University Hospital Basel, University of Basel, Petersgraben 4, 4031 Basel, Switzerland; Department of Cardiology, University Hospital Basel, University of Basel, Petersgraben 4, 4031 Basel, Switzerland; Cardiovascular Research Institute Basel, University Hospital Basel, University of Basel, Petersgraben 4, 4031 Basel, Switzerland; Department of Cardiology, University Hospital Basel, University of Basel, Petersgraben 4, 4031 Basel, Switzerland; Cardiovascular Research Institute Basel, University Hospital Basel, University of Basel, Petersgraben 4, 4031 Basel, Switzerland; Department of Cardiology, University Hospital Basel, University of Basel, Petersgraben 4, 4031 Basel, Switzerland; Cardiovascular Research Institute Basel, University Hospital Basel, University of Basel, Petersgraben 4, 4031 Basel, Switzerland; Department of Cardiology, University Hospital Basel, University of Basel, Petersgraben 4, 4031 Basel, Switzerland; Cardiovascular Research Institute Basel, University Hospital Basel, University of Basel, Petersgraben 4, 4031 Basel, Switzerland; Department of Cardiology, University Hospital Basel, University of Basel, Petersgraben 4, 4031 Basel, Switzerland; Cardiovascular Research Institute Basel, University Hospital Basel, University of Basel, Petersgraben 4, 4031 Basel, Switzerland; Department of Cardiology, University Hospital Basel, University of Basel, Petersgraben 4, 4031 Basel, Switzerland; Cardiovascular Research Institute Basel, University Hospital Basel, University of Basel, Petersgraben 4, 4031 Basel, Switzerland

**Keywords:** Pulsed-field ablation, Pulmonary vein isolation, Pericarditis, Atrial fibrillation

## Abstract

**Graphical Abstract:**
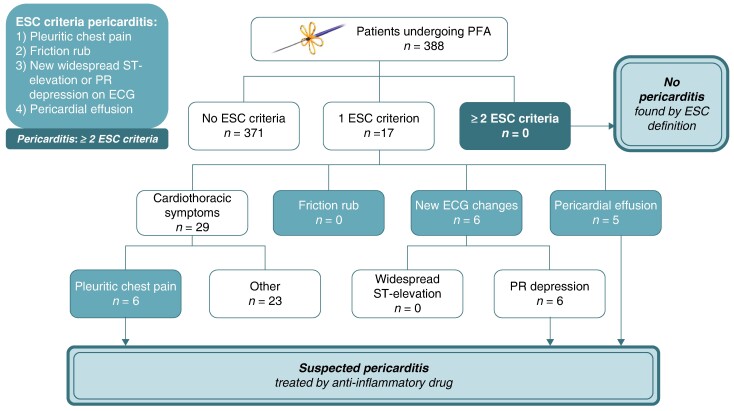

Pulsed-field ablation (PFA) has emerged as a novel treatment strategy for patients with atrial fibrillation (AF). This new ablation approach utilizes high-energy pulsed electric fields to induce tissue necrosis and cell death. Adequately dosed PFA provides tissue selectivity for myocardium and showed a promising safety profile in previous studies.^1–3^ While traditional thermal ablation modes such as radiofrequency (RF) energy or cryoballoon (CB) energy showed an acute pericarditis rate of 0.8–10.2%,^4^ little is known about the incidence of acute pericarditis after PFA. The aim was to assess the incidence of acute pericarditis using PFA for the treatment of AF.

Patients undergoing catheter ablation for AF using PFA were consecutively enrolled in a prospective, observational registry in a tertiary referral centre between January 2022 and January 2024. Pulsed-field ablation was performed using a pentaspline catheter system (FARAPULSE, Boston Scientific, Natick, MA, USA) as previously described.^1–3^ After PFA, all patients received a standardized, comprehensive assessment for the presence of acute pericarditis including clinical assessment for cardiothoracic symptoms, cardiac auscultation, 12-lead electrocardiogram (ECG), and transthoracic echocardiography. Furthermore, a standardized follow-up was performed in all patients at 3, 6, and 12 months. A clinical diagnosis of acute pericarditis was established according to the 2015 European Society of Cardiology (ESC) guidelines^5^ by the presence of at least two of the following criteria within 90 days after PFA: (i) pleuritic chest pain; (ii) friction rub; (iii) new widespread ST-elevation or PR depression on ECG; and (iv) pericardial effusion. Suspected acute pericarditis was diagnosed if one criterion was fulfilled.

A total of 388 patients (median age 68 years, 32% female) underwent pulmonary vein isolation (PVI) using PFA for the treatment of AF. Among them, 349 patients (90%) received a treatment with PFA only, and in 39 patients (10%), an additional cavotricuspid isthmus ablation using RF energy was performed. A PVI-only protocol was implemented in 223 patients (67%), and 128 patients (33%) received additionally posterior wall isolation. Only 24 patients (6.2%) received more than 50 applications.

The median number of applications was 32 [Interquartile range (IQR) 20–35] applications, and 302 patients (78%) underwent first ablation, while 86 patients (22%) had a redo procedure.

Zero patients (0%) fulfilled ≥2 ESC criteria required for diagnosis of acute pericarditis. Suspected acute pericarditis within 90 days after the procedure was found in 17 patients (4.4%), with 16 patients having PFA-only and one patient having additional cavotricuspid isthmus ablation performed using RF energy. Twenty-nine patients (7.5%) reported some sort of cardiothoracic symptoms (chest pain, chest tightness, chest discomfort, shortness of breath, or pleuritic chest pain). Among them, six patients (1.5%) received drug treatment due to suspected pericarditis. Zero patients (0%) had a friction rub, six patients (1.5%) showed PR depression (no ST-elevation), and five patients (1.3%) demonstrated new post-procedural pericardial effusions (*Graphical Abstract*). None of the pericardial effusions required drainage. We found no trend of fewer suspected pericarditis cases over time, as assessed by the Cochran–Armitage test for trend (*P* = 0.1999). A statistical analysis using logistic regression showed no correlation between the occurrence of one ESC criterion and the number of PFA applications during PVI (*P* = 0.935).

Our findings corroborate and extend previous findings regarding the presence of acute pericarditis after PFA for the treatment of AF. The MANIFEST-PF registry^1^ assessed 1568 patients among 24 centres undergoing PFA and reported one (0.06%) case of acute pericarditis. Similarly, in the multi-centre EU-PORIA registry^2^ containing 1233 patients, acute pericarditis occurred in two patients (0.16%). Finally, in the ADVENT randomized clinical trial,^3^ 607 AF patients were 1:1 randomized to PFA or RF/CB ablation. Acute pericarditis occurred in one patient (0.3%).

We acknowledge several limitations. This is a single-centre study with all its limitations. Due to a possibly very low incidence rate, the sample size might not be large enough to detect rare cases of post-ablation pericarditis after PFA. Furthermore, larger studies are warranted. Transseptal puncture was not routinely guided by intra-cardiac echocardiography, which minimizes the rate of pericardial effusion due to these procedural steps.

In this large cohort of patients undergoing PVI with a pentaspline PFA platform using the recommended workflow, no patients suffered acute pericarditis according to current ESC criteria. Any type of cardiothoracic symptoms after PFA was noted in 7.5% of patients with 1.5% of patients requiring drug therapy.

## Data Availability

The data underlying this article will be shared on reasonable request to the corresponding author.
